# Evidence for Ongoing Modeling-Based Bone Formation in Human Femoral Head Trabeculae *via* Forming Minimodeling Structures: A Study in Patients with Fractures and Arthritis

**DOI:** 10.3389/fendo.2018.00088

**Published:** 2018-03-19

**Authors:** Hiroshige Sano, Naoki Kondo, Taketoshi Shimakura, Junichi Fujisawa, Yasufumi Kijima, Tomotake Kanai, Kenneth E. S. Poole, Noriaki Yamamoto, Hideaki E. Takahashi, Naoto Endo

**Affiliations:** ^1^Division of Orthopedic Surgery, Department of Regenerative and Transplant Medicine, Niigata University Graduate School of Medical and Dental Sciences, Niigata, Japan; ^2^Niigata Bone Science Institute, Niigata, Japan; ^3^Department of Medicine, University of Cambridge, Cambridge, United Kingdom; ^4^Department of Orthopedic Surgery, Niigata Rehabilitation Hospital, Niigata, Japan

**Keywords:** modeling-based bone formation, forming minimodeling structures, femoral head, bone histomorphometry, femoral neck fracture, rheumatoid arthritis, hip osteoarthritis

## Abstract

Bone modeling is a biological process of bone formation that adapts bone size and shape to mechanical loads, especially during childhood and adolescence. Bone modeling in cortical bone can be easily detected using sequential radiographic images, while its assessment in trabecular bone is challenging. Here, we performed histomorphometric analysis in 21 bone specimens from biopsies collected during hip arthroplasty, and we proposed the criteria for histologically identifying an active modeling-based bone formation, which we call a “forming minimodeling structure” (FMiS). Evidence of FMiSs was found in 9 of 20 specimens (45%). In histomorphometric analysis, bone volume was significant higher in specimens displaying FMiSs compared with the specimens without these structures (BV/TV, 31.7 ± 10.2 vs. 23.1 ± 3.9%; *p* < 0.05). Osteoid parameters were raised in FMiS-containing bone specimens (OV/BV, 2.1 ± 1.6 vs. 0.6 ± 0.3%; *p* < 0.001, OS/BS, 23.6 ± 15.5 vs. 7.6 ± 4.2%; *p* < 0.001, and O.Th, 7.4 µm ± 2.0 vs. 5.2 ± 1.0; *p* < 0.05). Our results showed that the modeling-based bone formation on trabecular bone surfaces occurs even during adulthood. As FMiSs can represent histological evidence of modeling-based bone formation, understanding of this physiology in relation to bone homeostasis is crucial.

## Introduction

Despite significant effort over recent decades, the histological finding of modeling-based bone formation on trabecular bone still remains elusive. Bone modeling is the biological process that shapes and sizes bone in response to physiological influences or mechanical forces encountered by the skeleton, and is essential especially during skeletal growth (1, 2) and reaction to mechanical loading (3, 4). In bone modeling, bone resorption and bone formation are not necessarily coupled in a site-specific manner, as they are in the process of remodeling-based bone formation (3–7).

Frost first proposed that the process of modeling-based bone formation could be identified in trabecular bone under the microscope, and termed this “minimodeling” (2, 8). Some authors have reported the phenomenon of minimodeling in postmenopausal women during teriparatide (PTH) treatment (9, 10), in uremic patients (11–15), and in rats treated with prostaglandin E2 (16) and vitamin D3 (17, 18). These were from the histological evidence of bone formation upon a smooth cement line. However, we propose that the definition of modeling-based bone formation should not rely on the characteristics of the cement line (smooth or scalloped). This is because cement lines are typically associated with the resorption of primary or secondary bone corresponding to the extent of osteoclastic Howship’s lacunae at the periphery of secondary osteons (19), which is not appropriate when considering the process of modeling-based bone formation.

In this study, we clarified the definition of the structure for modeling-based bone formation and defined this histological appearance as a “minimodeling structure” (MiS), because the original description of minimodeling referred to the process of bone formation (2). We further proposed the term “forming minimodeling structure” (FMiS) for the MiS covered with an osteoid seam, which represents an active state in bone formation (20). We then performed histomorphometric analyses in bone biopsies from the femoral heads of patients with femoral neck fracture (FN), rheumatoid arthritis (RA), and osteoarthritis (OA). In particular, we investigated bone histomorphometric parameters in specimens with and without FMiSs to evaluate the histological differences between these groups, which could provide a better understanding of the underlying mechanism of trabecular bone modeling.

## Materials and Methods

### Specimens

Our analysis was performed on 21 femoral heads obtained from 20 Japanese adults who underwent total hip arthroplasty or femoral head replacement at Niigata University Hospital and Niigata Rehabilitation Hospital. Written informed consent was obtained from each patient in accordance with the ethics committee of Niigata University Medical and Dental Hospital, and Niigata Rehabilitation Hospital. This study was in compliance with the standard guidelines for human research (Declaration of Helsinki).

### Undecalcified Histology

**Figure 1:**
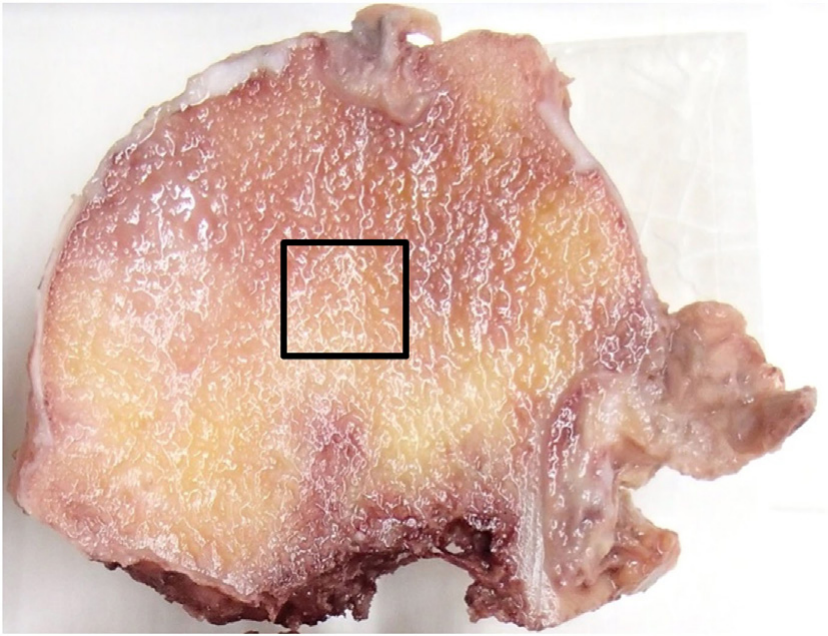
Specimen preparation. A bone block of 10 mm × 10 mm × 10 mm was excised from the central region of the femoral head as indicated by the squared area.

After fixing with 70% ethanol, a bone block of 10 mm × 10 mm × 10 mm was excised from the central region of the femoral head, encompassing purely trabecular bone (Figure 1). Villanueva bone staining was performed according to a previously reported protocol (21). Thereafter, the excised tissue was dehydrated in ascending grades of ethanol and acetone, and subsequently infiltrated and embedded in methylmethacrylate without decalcification. The resulting blocks were then sectioned at a thickness of 5 µm by microtome (Leica RM2255). A single 10 mm × 10 mm section from the sagittal midplane was selected from each sample.

### Quantitative Conventional Static Histomorphometry

Bone histomorphometric measurements were performed as previously described (7, 22, 23). Briefly, the bone structural unit was identified under the polarized light microscope. Measurements were then performed using a semiautomatic image analyzing system (Histometry RT CAMERA, System Supply, Nagano, Japan) with a 20× objective lens and one set of 10× oculars, and at 200× magnification. Analyses of the structural parameters [bone volume/tissue volume (BV/TV, %), trabecular thickness (Tb.Th, μm)], the static formation parameters [osteoid volume/bone volume (OV/BV, %), osteoid surface/bone surface (OS/BS, %), osteoid thickness (O.Th, μm)], and the static resorption parameter [eroded surface/bone surface (ES/BS, %)] were carried out according to the standards of the American Society for Bone and Mineral Research (24, 25).

### Forming Minimodeling Structure

**Figure 2:**
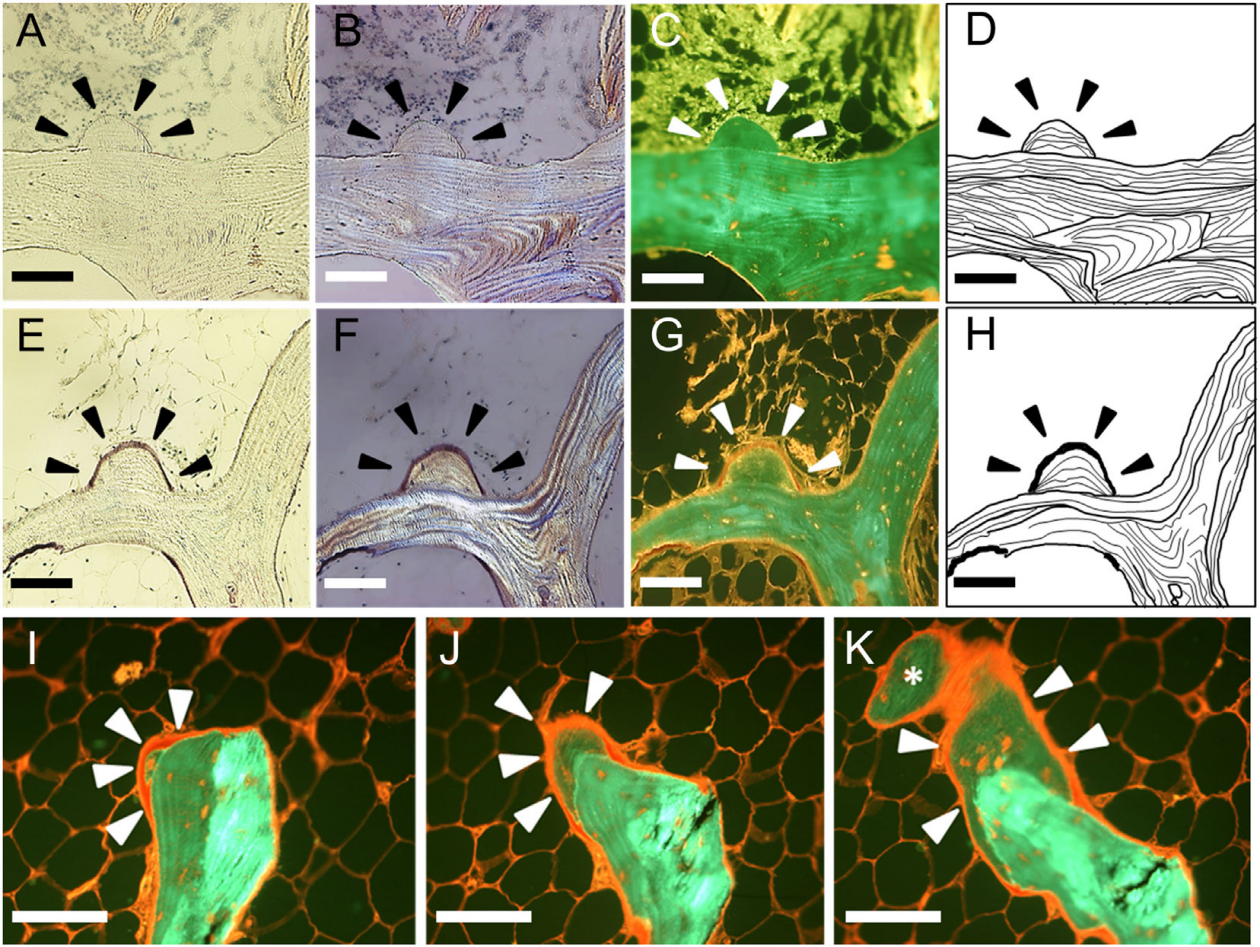
Histological findings in minimodeling with and without osteoid seam. A minimodeling structure (MiS) without an osteoid seam **(A–D)**. Arrowheads indicate the representative MiS. The base of the MiS is a smooth lamellar bone surface without any evidence of previous osteoclastic Howship’s lacunae. The lamellar pattern of the base is different from that of the MiS. A forming minimodeling structure (FMiS) **(E–H)**. An osteoid seam was detected on the MiS surface as purple under polarized light microscopy [**(F)**, arrowheads], and red under fluorescent microscopy [**(G)**, arrowheads]. The degree of fluorescent signal in the FMiS was lower than that of the base **(G)**. An analysis of consecutive sections [**(I–K)**; I → J → K]. An FMiS was detected on the smooth bone surface without evidence of previous bone resorption **(I)**, which showed growth **(J)** and connection to the adjacent trabecular bone as indicated by an asterisk **(K)**. Bright-field microscopy **(A,E)**; polarized light microscopy **(B,F)**; fluorescent microscopy **(C,G,I–K)**; diagram of the MiS **(D,H)**. Scale bars: 100 µm.

We referred to the histological finding of the modeling-based bone formation on trabecular bone with no evidence of previous resorption as a MiS. We proposed the following histological criteria for the definition of the MiS: (a) the base of the MiS is a smooth lamellar bone surface, and (b) the lamellar pattern of the MiS is different from that of the base of the bone surface (Figures 2A–D). We suggested the following histological criteria to define a “FMiS” (Figures 2E–H): (a) the surface of the MiS is covered with an osteoid seam of at least 3-µm thickness under polarized light microscopy (Figure 2F), and (b) the degree of fluorescent signal in bone in the MiS is different and lower than that of older adjacent bone as observed in Figure 2G (arrowheads). This is due to its relatively lower mineralization, which was confirmed by comparison with contact micro-radiograph (Figure S1 in Supplementary Material). To visualize the FMiS in 3D, we performed an additional analysis of 30 consecutive sections in 1 specimen with evident FMiSs, focusing on the FMiS. In histomorphometric analysis, we investigated the number of FMiSs/total bone surface (N.FMiS/μm), and the bone volume [BV (FMiS)] and bone surface [BS (FMiS)] of FMiSs in each FMiS-containing specimen. Using the result of conventional static histomorphometry, BV (FMiS)/BV (%), BV (FMiS)/OV (%), BS (FMiS)/BS (%), and BS (FMiS)/OS (%) were calculated.

### Analysis of the Specimens from Patients

Age, body mass index (BMI), diagnosis for hip surgery, therapies for osteoporosis (OP) and RA, respectively, were analyzed in each patient. In patients with RA, we also analyzed disease activity score (DAS) 28-ESR3 (26, 27), blood serum level of C-reactive protein (CRP) and matrix metalloproteinase-3 (MMP-3), and dose of prednisolone (PSL). We conducted three analyses with conventional static histomorphometry: (a) comparison between the specimens with (*n* = 9) and without FMiSs (*n* = 11), (b) comparison between the RA specimens with (*n* = 7) and without FMiSs (*n* = 6), and (c) among specimens from patients with RA who had hip surgery due to joint destruction, comparison between these with (*n* = 6) and without FMiSs (*n* = 3).

### Statistical Analysis

Mann–Whitney sum rank tests were used. Data are expressed as the mean ± SD. All the analyses were performed by using GraphPad Prism software (GraphPad, La Jolla, CA, USA). *p*-Value < 0.05 was considered statistically significant.

## Results

### Characteristics of the Patients

This study included 21 specimens from 20 patients (Table 1). Of these, 1 patient underwent bilateral hip surgery (Case 3, No. 14, 15), and 1 femoral head specimen from a male patient with RA was excluded from our analysis due to the unreliability of the histomorphometric analysis from the extent of joint destruction (Case 9, No. 21). Patients included in the analysis ranged from 27 to 90 years of age (mean ± SD, 69.3 ± 16.2 years), with 16 women (84%) and 3 men (16%). There were eight patients using bisphosphonate therapies for OP (No. 2, 3, 5, 6, 13, and 18–20). Among 13 specimens from 12 patients with RA, 4 specimens were from femoral head replacement as a treatment for FN (No. 1, 3, 5, and 17), and 9 were from total hip arthroplasty performed due to RA-related destruction of the hip joint (No. 2, 4, 6, 13–16, 19, and 20). The remaining seven specimens were obtained from FN in patients with OP (No. 8–11 and 18), trauma (No. 7), and secondary OA due to a traumatic hip joint dislocation (No. 12).

**Table 1:** Characteristics of the patients.

FMiS-negative group							FMiS-positive group						
					Therapies for							Therapies for	
Case	No.	Sex	Age	Diagnosis	OP	RA	Case	No.	Sex	Age	Diagnosis	OP	RA
1	1	F	64	RA, FN		MTX, TAC	1	12	F	27	OA; trauma		
2	2	F	87	RA, OP	BP	BUC	2	13	F	43	RA, OP	BP	ETN, MTX, PSL
3	3	F	76	RA, FN, OP	BP	MTX, SASP	3	14	F	57	RA		TCZ
4	4	M	78	RA		BUC, PSL	3	15	F	57	RA		TCZ
5	5	F	77	RA, FN, OP	BP	SASP, PSL	4	16	F	62	RA		PSL
6	6	F	55	RA, OP	BP	MTX, BUC, PSL	5	17	F	73	RA, FN		PSL
7	7	M	56	FN			6	18	F	77	FN, OP	BP	
8	8	F	86	FN, OP	Vit.D		7	19	F	80	RA, OP	BP	BUC, PSL
9	9	F	75	FN, OP			8	20	F	82	RA, OP	BP	ETN, MTX, PSL
10	10	M	83	FN, OP			9	21	M	60	RA; excluded		MTX
11	11	F	90	FN, OP									

*FMiS, forming minimodeling structure; No., number of specimens; F, female; M, male; RA, rheumatoid arthritis; FN, femoral neck fracture; OA, osteoarthritis; OP, osteoporosis; BP, bisphosphonate; Vit.D, vitamin D; MTX, methotrexate; TAC, tacrolimus; BUC, bucillamine; SASP, sulfasalazine; PSL, prednisolone; IFX, infliximab; ETN, etanercept; TCZ, tocilizumab*.

### Histomorphometric Data in FMiS-Positive and -Negative Groups

At least 1 FMiS was identified in 9 of our 20 specimens (45%), and we called this group FMiS-positive. These nine specimens were from eight individuals. The analysis of consecutive sections among one of this group showed that the FMiS grew and connected to the adjacent trabecular bone (Figures 2I–K; Video S1 in Supplementary Material).

**Figure 3:**
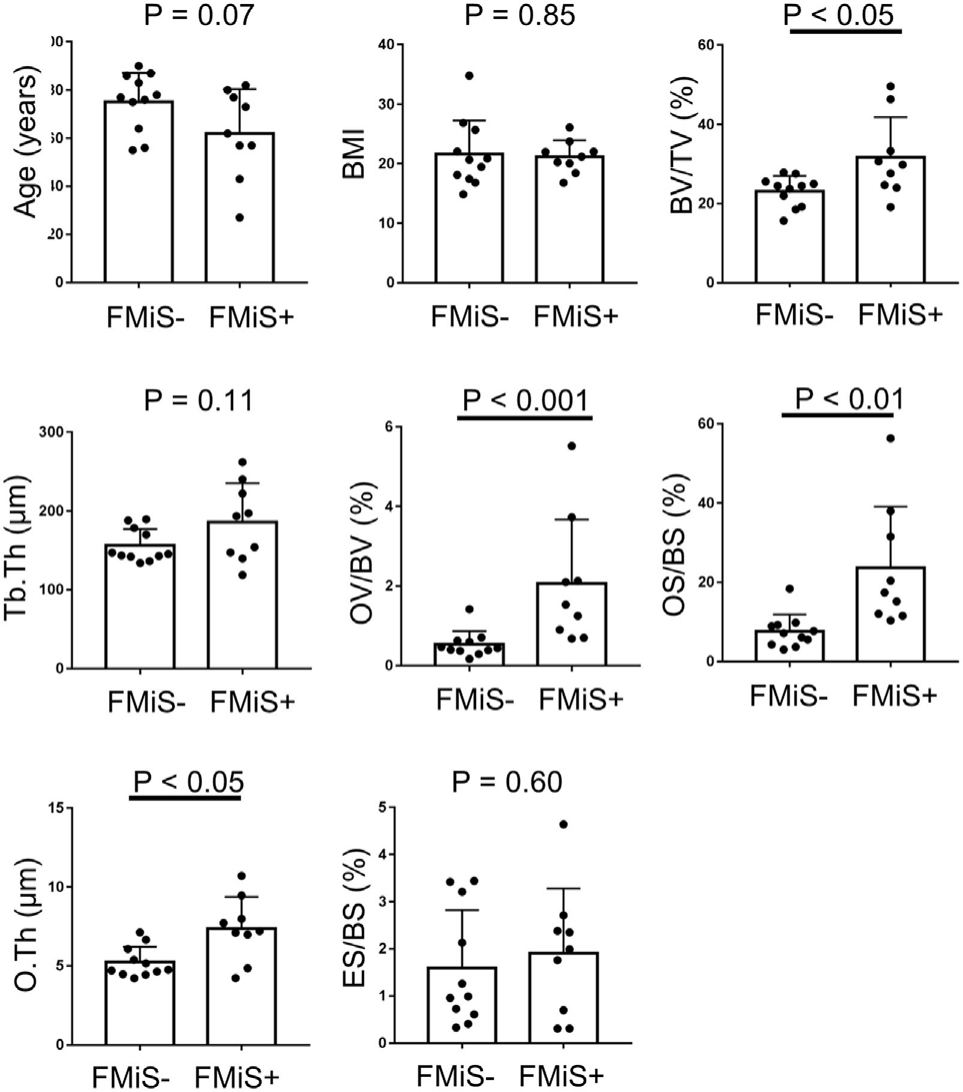
Comparison between FMiS-positive and -negative specimens. FMiS-positive group (FMiS+) showed significantly higher values in bone histomorphometric data (BV/TV, OV/BV, OS/BS, and O.Th) compared with FMiS-negative group (FMiS−). Data are mean ± SD. Abbreviations: FMiS, forming minimodeling structure; BMI, body mass index; BV, bone volume; TV, tissue volume; Tb.Th, trabecular thickness; OV, osteoid volume; BV, bone volume; OS, osteoid surface; BS, bone surface; O.Th, osteoid thickness; ES, eroded surface.

There were no significant differences in age (62.0 ± 18.4 vs. 75.2 ± 12.0; *p* = 0.12) and BMI (21.2 ± 2.8 vs. 23.3 ± 6.5; *p* = 0.90) between FMiS-positive and -negative groups. In histomorphometric data, the FMiS-positive group showed that BV/TV and the static formation parameters were significantly higher than those of FMiS-negative group [BV/TV 31.7 ± 10.2 vs. 23.1 ± 3.9% (*p* < 0.05); OV/BV, 2.1 ± 1.6 vs. 0.6 ± 0.3% (*p* < 0.001); OS/BS, 23.6 ± 15.5 vs. 7.6 ± 4.2% (*p* < 0.001); and O.Th, 7.4 ± 2.0 vs. 5.2 ± 1.0 µm (*p* < 0.05)]. However, there were no significant differences between FMiS-positive group and -negative group in Tb.Th (186.1 ± 49.2 vs. 156.2 ± 21.0 µm; *p* = 0.11) and ES/BS (1.9 ± 1.4 vs. 1.6 vs. 1.2%; *p* = 0.86) (Figure 3). The N.FMiS and N.FMiS/BS ranged from 1 to 10 and 2.1 × 10^−6^ to 3.0 × 10^−5^/μm, respectively, which did not show significant correlation with histological variables of osteoid parameters (OV/BV, OS/BS, and O.Th) in FMiS-positive group (data not shown). The average values of BV (FMiS)/BV and BS (FMiS)/BS were 0.1% (1.7 × 10^−2^ to 0.2%) and 0.8% (0.1–1.5%), while the average values of BV (FMiS)/OV and BS (FMiS)/OS were 5.4% (0.7–9.1%) and 3.8% (1.4–10.9%), respectively.

### Histomorphometric Data in RA Specimens

**Figure 4:**
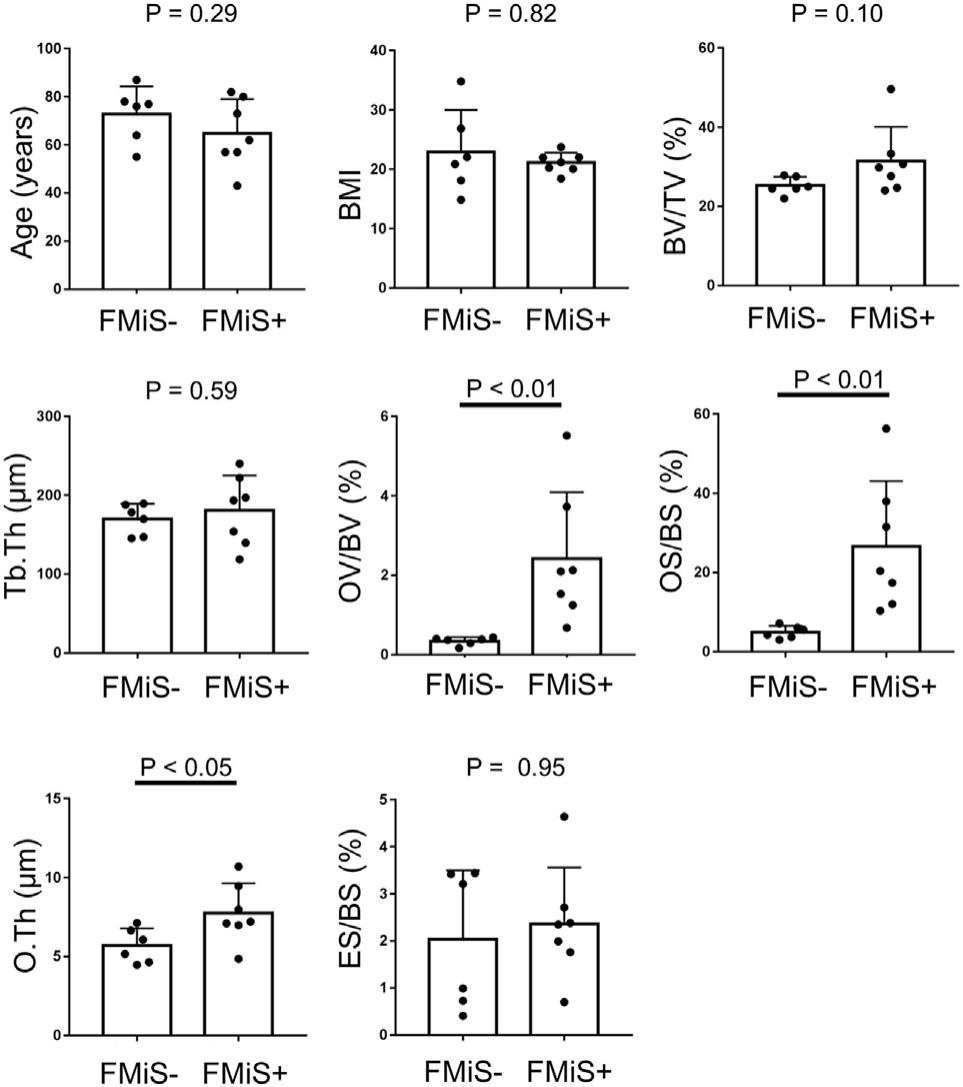
Comparison between FMiS-positive and -negative specimens from patients with RA. FMiS-positive (FMiS+) group showed significantly higher values in bone histomorphometric data (OV/BV, OS/BS, and O.Th) compared with FMiS-negative group (FMiS−). Data are mean ± SD. Abbreviations: FMiS, forming minimodeling structure; BMI, body mass index; BV, bone volume; TV, tissue volume; Tb.Th, trabecular thickness; OV, osteoid volume; BV, bone volume; OS, osteoid surface; BS, bone surface; O.Th, osteoid thickness; ES, eroded surface.

Forming minimodeling structures were identified in 7 of our 13 specimens (54%) from 6 of 12 individuals. Comparing FMiS-positive with -negative groups, there were no significant differences in age (64.9 ± 14.1 vs. 72.8 ± 11.4; *p* = 0.42), BMI (21.1 ± 1.7 vs. 22.9 ± 7.1; *p* = 0.82), DAS28-ESR3 (2.9 ± 0.5 vs. 3.1 ± 1.0; *p* = 0.42), CRP (0.6 ± 0.7 vs. 0.6 ± 0.7 mg/dL; *p* = 0.81), MMP-3 (136.0 ± 115.4 vs. 170.9 ± 186.3 ng/mL; *p* = 0.99), and PSL intake (5.0 ± 5.2 vs. 2.7 ± 3.9; *p* = 0.99). In histomorphometric data, FMiS-positive group showed that statistic formation parameters were significantly higher than those of the FMiS-negative group: OV/BV, 2.4 ± 1.7 vs. 0.3 ± 0.1% (*p* < 0.01); OS/BS, 26.6 ± 16.5 vs. 5.0 ± 1.6% (*p* < 0.01); and O.Th, 7.8 ± 1.9 vs. 5.7 ± 1.1 µm (*p* < 0.05). However, there were no significant differences between FMiS-positive and -negative groups in BV/TV (31.4 ± 8.7 vs. 25.3 ± 2.2%; *p* = 0.10), Tb.Th (180.8 ± 44.6 vs. 169.8 ± 19.5 µm; *p* = 0.53), and ES/BS (2.4 ± 1.2 vs. 2.0 vs. 1.5%; *p* = 0.95) (Figure 4). Six out of 7 in FMiS-positive specimens showed that the reason for hip surgery was hip joint destruction (86%). Of the 9 RA specimens from hip joint destruction patients, 6 (67%) were positive for FMiSs (Table 1). The comparison between this FMiS-positive (*n* = 6) and -negative groups (*n* = 3) showed that OV/BV (1.7 ± 1.5 vs. 0.4 ± 0.1%; *p* < 0.05) and OS/BS (29.3 ± 16.3 vs. 5.5 ± 1.7%; *p* < 0.05) were significantly increased in FMiS-positive group, while there were no statistically significant differences in age, BMI, DAS28-ESR3, CRP, MMP-3, PSL intake, BS, BV, TV, BV/TV, Tb.Th, O.Th, and ES/BS (data not shown).

## Discussion

The establishment of a definition for the histological finding of modeling-based bone formation is important for evaluating bone histology. Here, we clarified the definition of minimodeling structures (MiS), and described new histological criteria for identifying them in their active forming state, which we now define as, “forming minimodeling structures” (FMiS). Trans iliac biopsies from postmenopausal women were previously examined for evidence of modeling-based bone formation, which we call MiSs in this study. The percentage of patients with MiSs varied from 0 to 63% (7, 9, 10), and in those treated with human PTH it was 0.4–40.0% (9, 10). This confirms a wide range probably due to the vague definition of minimodeling in histological findings and the difficulty in MiSs to be distinguished from the old lamellar bone. In contrast the demarcation of an FMiS which is covered with an osteoid seam is clear. Therefore, an important aspect of the present paper is to define these FMiSs, where ongoing surface osteoid gives additional information on forming surfaces. Our findings identified the presence of FMiSs in 9 out of 20 specimens (45%), providing strong evidence for modeling-based bone formation on trabeculae in loaded femoral head bone even in the elderly. Moreover, we demonstrated that FMiSs were highly prevalent in specimens with high static formation parameters. The cause of hip surgery in FMiS-positive was joint destruction in 7 out of 9 (78%) in total specimens, and 6 out of 7 (86%) in RA specimens (Table 1). This suggests that the appearance of FMiSs may associate more with joint destruction than with femoral neck fracture. Therefore, we conducted the subsequent analysis in specimens from patients with RA who had hip surgery due to joint destruction. Again these results showed higher values of OV/BV and OS/BS in FMiS-positive than in -negative group. FMiS-positive specimens showed that the average of BV (FMiS)/OV and BS (FMiS)/OS were 5.4 and 3.8%, which were 50 times and 4 times higher in that of BV (FMiS)/BV and BS (FMiS)/BS, respectively. This was also compatible with FMiSs being associated with higher values of osteoid parameters.

This finding of FMiSs is quite different from that seen in pathological conditions such as osteomalacia (28, 29) or hypophosphatasia (30); all of our specimens showed a lamellar pattern in the FMiS itself, distinct from the underlying trabecular bone. The histological analysis revealed the normal range of osteoid thickness (<12 µm), and no scalloped appearance in specimens with FMiSs.

The analysis of consecutive sections with FMiS-positive clearly showed that new bone grew from a bone surface without previous bone resorption and connected with the adjacent trabecular bone (Figures 2I–K; Video S1 in Supplementary Material). We consider this connection process is the mechanism for the increase in connectivity as an adaptation to increased mechanical stimuli, as others hypothesized for the process of minimodeling (2, 31, 32). Although we did not measure the connectivity of trabecular bone in each sample with μCT images (33, 34) in this study, Kazama et al. (35) reported that trabecular bone volume (BV/TV) is correlated with trabecular bone connectivity. In our study, FMiS-positive group showed a high value of BV/TV compared with -negative group (Figure 3) and a tendency in RA specimens (Figure 4), indicating FMiS-positive specimens are associated with an increase in connectivity of trabecular bone.

How the process of FMiSs is regulated still remains unclear. Among nine specimens from patients with RA who underwent total hip replacement, 67% showed the modeling-based bone formation on trabecular bone surfaces (No. 13–16, 19, 20). Conversely, among five specimens from elderly patients with low impact hip fracture (No. 8–11, 18), only one specimen showed FMiSs (20%; No. 18) (Table 1). We did not observe statistical differences in age and BMI between these two groups (63.5 ± 15.0 vs. 78.0 ± 13.5; *p* = 0.18, 21.3 ± 2.0 vs. 20.0 ± 3.5; *p* = 0.19, respectively). Whether inflammatory arthritis is associated with ongoing modeling-based bone formation in the central femoral head is a challenging issue that needs to be addressed through a larger study of specimens from RA and non-arthritic conditions. Various functions of osteocytes in bone metabolism have been revealed so far (36–40). There is accumulating evidence of the involvement of sclerostin in the anabolic response within trabecular bone, which is regulated by the mechanical loading through the reduction of sclerostin positive osteocytes (41, 42). From the analysis of serum bone turnover markers, the anti-sclerostin antibody has been reported to increase bone formation *via* a process of modeling-based bone formation (43, 44). Although speculative, it is possible that the decrease in expression of sclerostin by osteocytes may play a key role in FMiSs. Nazarian et al. showed that femoral head trabeculae are highly loaded in habitual daily activities (45), however, it remains to be elucidated how such high loading conditions translate into building FMiSs.

The reason why N.FMiS/BS did not show significant correlation with the histological variables of osteoid parameters in the FMiS-positive group is probably due to the small number of specimens. We cannot exclude the possibility that FMiS would be a peripheral part of the remodeling-based bone structure, already reported as “spill over” (10), or of a mature phase of micro callus (46–51). To consider these theories, studies with larger numbers and use of consecutive sections are necessary. Other weaknesses of the present study include the lack of fluorescent double labels for assessing dynamic formation parameters, and of data about serum bone turnover markers (CTX and P1NP), 25(OH)D, and PTH as well as bone mineral density in each patient. The values of O.Th in our specimens were all within normal range, unlike osteomalacia, it would be useful to know 25(OH)D and PTH levels in order to exclude the possibility of mild insufficiency and secondary hyperparathyroid effects (52). Despite these limitations, we were able to confirm the presence of the FMiSs in individuals with higher values of static bone formation parameters. Further studies are needed to address the spatial location of the FMiSs with relation to their physiological and pathological microenvironment.

Our findings provide further evidence that modeling-based bone formation on trabecular bone, which is different from the process of remodeling-based bone formation, continues even in the elderly, and that FMiSs can be a predictive indicator in bone specimens for higher values of osteoid parameters.

## Ethics Statement

Ethics Committee at Niigata University School of Medicine approved this research on January 4, 2012 (Protocol Identification Number 1345), and Ethics committee of Niigata Rehabilitation Hospital approved this protocol on February 20, 2012 at the fifth 2012 Ethics committee.

## Author Contributions

All contributing authors have agreed to submission of this manuscript for publication. HS, NK, NY, HT, and NE conceived and designed the study. HS, NK, TS, JF, YK, TK, and NY performed experiments. HS, NK, TS, and YK analyzed data. HS, KP, NY, HT, and NE interpreted results and wrote the manuscript.

## Conflict of Interest Statement

The authors declare that the research was conducted in the absence of any commercial or financial relationships that could be constructed as a potential conflict of interest.

## Supplementary Material

Figure S1 Comparison with contact radio micrograph. A figure of contact radio micrograph from the half cross-section of the second metatarsal bone, which was collected from a 72-year-old woman who underwent osteotomy due to hallux valgus deformity (100 μm in thickness with a polished section) **(A)**. A figure of fluorescent microscopy after Villanueva bone staining from an adjacent bone area (25 μm in thickness with a polished section) **(B)**. Low mineralization area (grey) in contact radio micrograph **(A)** corresponds with low fluorescent signal (green) area in secondary osteon by fluorescent microscopy analysis **(B)**. Scale bar: 300 μm.

Video S1 Reconstructive video of FMiS from 30 consecutive sections. This video is a representation of the FMiS from 30 serial sections under fluorescence microscopy, which shows the FMiS grows and connects to adjacent trabecular bone to form a bridge. This video is from the same specimens as Figures 1I–K.
