# Supplementary figures and images for: Evidence for Ongoing Modeling-Based Bone Formation in Human Femoral Head Trabeculae via Forming Minimodeling Structures: A Study in Patients with Fractures and Arthritis
Source: Front Endocrinol (Lausanne). 2018 Mar 19;9:88. doi: 10.3389/fendo.2018.00088 (PMC5868326; doi:10.3389/fendo.2018.00088)

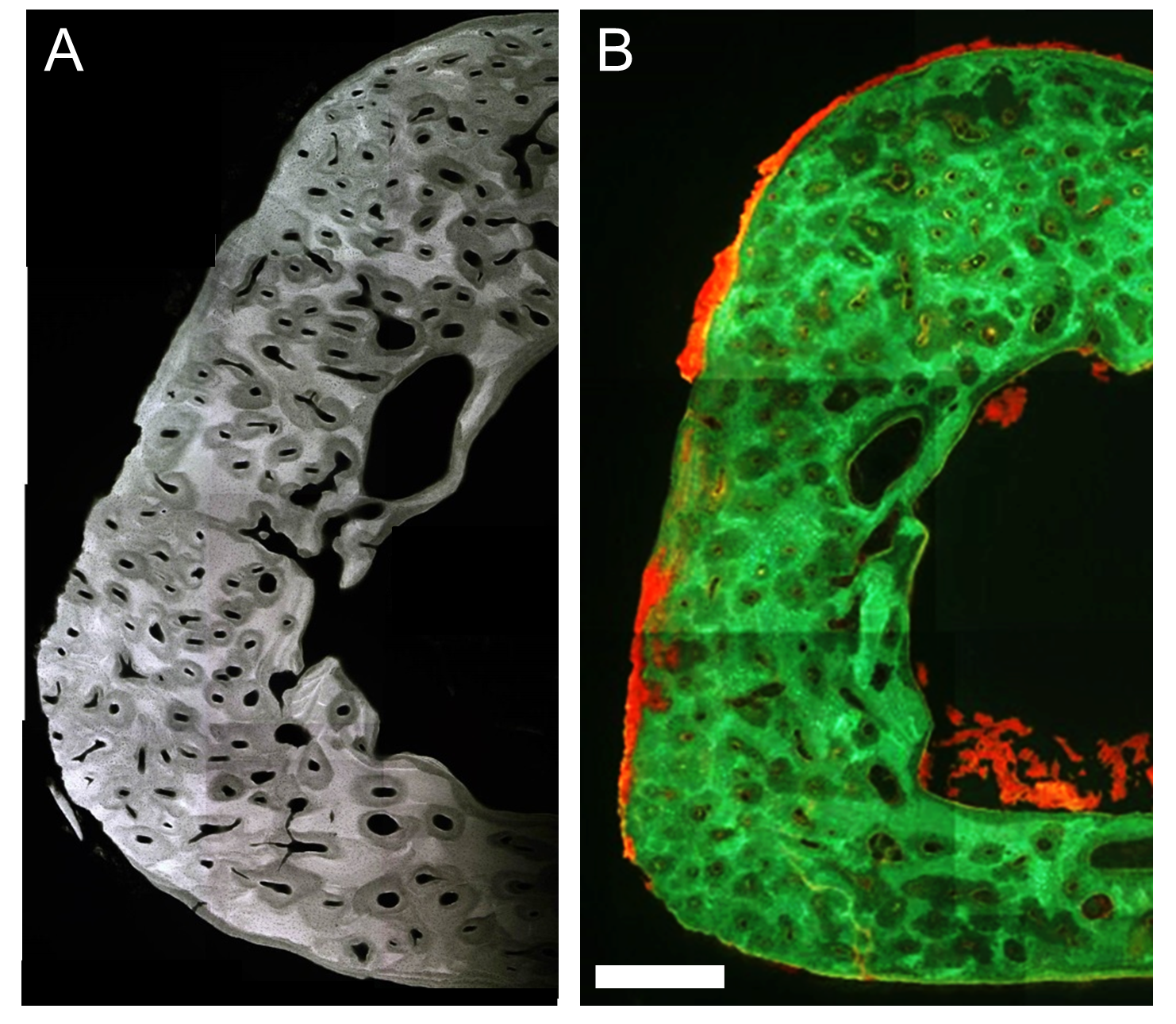

Supplement: Figure S1 — Comparison with contact radio micrograph. A figure of contact radio micrograph from the half cross-section of the second metatarsal bone, which was collected from a 72-year-old woman who underwent osteotomy due to hallux valgus deformity (100 μm in thickness with a polished section) (A). A figure of fluorescent microscopy after Villanueva bone staining from an adjacent bone area (25 μm in thickness with a polished section) (B). Low mineralization area (grey) in contact radio micrograph (A) corresponds with low fluorescent signal (green) area in secondary osteon by fluorescent microscopy analysis (B). Scale bar: 300 μm. [file image_1.tif]
